# Effectiveness of Zhong‐Yong thinking based dialectical behavior therapy group skills training versus supportive group therapy for lowering suicidal risks in Chinese young adults: A randomized controlled trial with a 6‐month follow‐up

**DOI:** 10.1002/brb3.1621

**Published:** 2020-04-18

**Authors:** Xueling Yang, Ding Liu, You Wang, Yu Chen, Weichen Chen, Caiyan Yang, Peining Zhang, Siyuan Ding, Xiaoyuan Zhang

**Affiliations:** ^1^ Department of Psychology Guangdong Provincial Key Laboratory of Tropical Disease Research School of Public Health Southern Medical University Guangzhou China; ^2^ Department of Psychiatry Zhujiang Hospital Southern Medical University Guangzhou China; ^3^ School of Nursing Southern Medical University Guangzhou China

**Keywords:** dialectical behavior therapy, randomized controlled trial, suicide prevention, Zhong‐Yong thinking

## Abstract

**Background:**

Dialectical behavior therapy (DBT) is a first‐line treatment for the prevention of suicide. Zhong‐Yong thinking could be viewed as a Chinese way of dialectical thinking, has long been a culturally dictating thinking style in China. To enhance cultural adaptability, we integrated Zhong‐Yong thinking into DBT group skills training and examined its efficacy in suicidal prevention compared with a supportive group therapy and a wait‐list group in high‐risk suicidal Chinese college students.

**Methods:**

A total of 97 suicidal participants were randomized to either Zhong‐Yong thinking based DBT group skills training (DBT_ZYT_, *n* = 33), or supportive group therapy (SGT; *n* = 32), or wait‐list group (WL; *n* = 32). DBT_ZYT_ was a 12‐week program based on Zhong‐Yong thinking instead of dialectical thinking, coaching participants mindfulness, emotion regulation, distress tolerance, and interpersonal effectiveness. Supportive group therapy was a 12‐week program aiming at improving interpersonal effectiveness and emotion regulation skills. Outcome measures were assessed at pre‐ and post‐treatment and 6‐month follow‐up.

**Results:**

At post‐treatment measures, the levels of suicidal ideation, hopelessness, psychache symptoms, and general psychopathology had significantly decreased in both intervention groups; at the 6‐month follow‐up measures, the intervention effects were better maintained in the DBT_ZYT_ group rather than in the SGT group. Specifically, DBT_ZYT_ was more effective in relieving participants’ long‐term obsessive‐compulsive, anxiety, hostility, phobic, psychotic, and additional symptoms.

**Conclusions:**

Zhong‐Yong thinking not only could integrate with DBT skills training in Chinese young adult population, but also has special strength in enhancing DBT’s efficacy.

## Graphical abstract

Zhong‐Yong thinking is a Chinese way of dialectical thinking Zhong‐Yong thinking based dialectical behavior therapy (DBT) lowered suicidal risks Zhong‐Yong thinking has special strength in enhancing DBT’s efficacy.

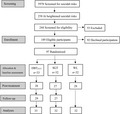

## 1 INTRODUCTION

Suicide is the second leading cause of death in young adults aged 15–29 years (WHO, 2017). The lifetime and 12‐month prevalence of suicidal thoughts and behaviors worldwide is 7.2% and 1.9% among college entrants (Mortier et al., 2018). There is 2.8% prevalence of suicide attempts and more than 600,000 suicide attempters among college students in China, indicating that more attention should be paid to this population (Yang, Zhang, Sun, Sun, & Ye, 2015).

Previous studies demonstrated that dialectical behavior therapy (DBT) could reduce suicidal risks by coaching participants mindfulness, distress tolerance, emotion regulation, interpersonal effectiveness, and self‐regulation skills (Fleischhaker et al., 2011; Linehan et al., 2015). DBT was initially developed for the treatment of borderline personality disorder, it is efficacy has received extensive empirical support in recent years (Bass, Van Nevel, & Swart, 2014; Linehan et al., 2006, 2015; Mcmain & Links, 2009; Panos et al., 2014). DBT is recognized as a first‐line treatment for the prevention of suicidal behavior in diverse clinical populations, including high‐risk and acutely suicidal clients (DeCou, Comtois, & Landes, 2018; Lin et al., 2019). The standard DBT includes individual psychotherapy, group skills training, telephone coaching, and a therapist consultation team. Among the shorter and less intensive versions of DBT, group skills training has received more attention and empirical supports (Gibson, Booth, Davenport, Keogh, & Owens, 2014; Krantz, Mcmain, & Kuo, 2018; Soler et al., 2009). The DBT group skills training incorporates almost all the important elements in the full DBT program, including mindfulness, emotion regulation, distress tolerance, and interpersonal effectiveness skills (Linehan, 1993).

The theoretical basis of DBT originates from dialectical thinking, which emphasizes that reality is comprised of two opposing forces or positions and intends to pursue a balance between two extremes and bring them together (Lynch, Chapman, Rosenthal, Kuo, & Linehan, 2010). Dialectical thinking is thus a process whereby apparent contradictions are able to be synthesized to provide a broader understanding of the “truth” in a situation. It seems that dialectical thinking should be easily accepted by easterners (Miyamoto & Ryff, 2011), given that Zhong‐Yong thinking has long been a culturally dictating thinking style in China, and some researchers argued that Zhong‐Yong thinking could be viewed as a Chinese way of dialectical thinking (Chiu, 2000; Liu, Wang, & Yang, 2015; Nisbett, Peng, Choi, & Norenzayan, 2001). However, DBT’s application in China is limited. Specifically, apart from a few literatures that introduce the practice of DBT, research investigating the treatment effects of DBT using randomized controlled trials in China is scarce. Therefore, we wonder that whether the effect of DBT intervention would be equivalent or superior to other widely‐used interventions in Chinese suicidal young adults? Is it necessary to consider cultural adaptability when applying DBT intervention to Chinese population?

Recent studies demonstrate that Zhong‐Yong (middle‐way) thinking still has indispensable influence on Chinese people's mental health (Yang et al., 2016) and behavioral performance (Pan & Sun, 2017; Yao, Yang, Dong, & Wang, 2010). Zhong‐Yong thinking, as the most representative eastern philosophy, derives from Confucianism, emphasizes the virtue of pursuing the middle ground and never going to extremes (Yang, 2009). Zhong‐Yong thinking is acknowledged as a metacognitive process, characterized by acting appropriately and flexibly under different situations. It has a profound influence on shaping Chinese people’ perception and cognition (Chang & Yang, 2014; Chiu, 2000; Guo, Li, Huang, & Chen, 2017; Kim, Yang, & Hwang, 2006; Wu & Lin, 2005). Theoretically, both dialectical and Zhong‐Yong thinking advocate tolerance of contradiction, avoiding going to extremes, and overcoming dichotomous, rigid patterns of thinking. In practice, these two thinking styles were both found to be beneficial for regulating extreme emotional distress (Miyamoto & Ryff, 2011; Yang et al., 2016). Therefore, it might be plausible to incorporate Zhong‐Yong thinking into DBT treatment of Chinese suicidal young adults. Moreover, compared with dialectical thinking, the concept of Zhong‐Yong thinking is more easily accepted and understood by Chinese people; and the connotation of Zhong‐Yong thinking is more compatible with the cognitive style of the Chinese. In the present study, we combined DBT group skills training with Zhong‐Yong thinking as the underlying philosophy to improve its cultural adaptability. Making such modification to the well‐established DBT practice among an eastern population whose thinking style is different from their western counterparts (Xinyue, Lingnan, Qing, Junpeng, & Baumeister, 2012) would further enhance the understanding of whether and how a psychological intervention could apply to individuals in different cultural contexts. In particular, the purposes of the present study were to explore whether DBT could integrate with Zhong‐Yong thinking in Chinese young adults with high‐suicidal risks, and to test whether Zhong‐Yong thinking based DBT would demonstrate superior or equivalent or inferior efficacy compared with supportive group therapy and wait‐list group.

## 2 MATERIALS AND METHODS

### 2.1 Study design and participants

We conducted a three‐arm randomized nonclinical controlled trial. We offered two interventions without fee: Zhong‐Yong thinking based DBT (DBT_ZYT_) group skills training and supportive group therapy (SGT), comparing the outcome measures with a wait‐list (WL) group. The length of treatment was relatively brief (12 weeks instead of the standard DBT skills training of 16 weeks), because participants showed less severe suicidal risks compared with clinical suicidal patients. The protocol was approved by the Ethics Committee of Southern Medical University.

**FIGURE 1:**
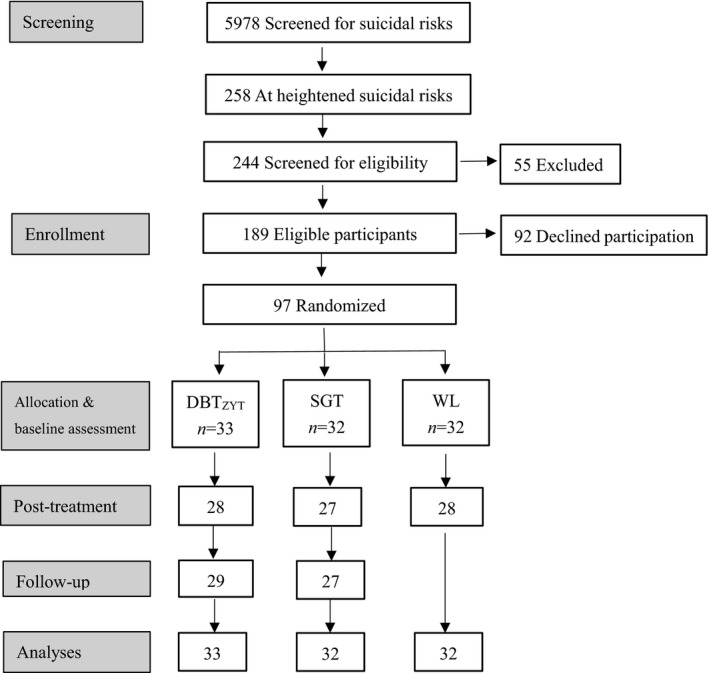
Participants flow diagram

Participants were 97 high‐suicidal risk college students recruited from Oct. 2015 to Dec. 2016. In the 2015 and 2016 school year, all the first‐year students (a total of 5,978 students) in Southern Medical University were screened using Suicidal Behaviours Questionnaire‐Revised (SBQ‐R) and other questionnaires measuring mental health. According to the SBQ‐R score, 258 students were identified as in heightened suicidal risks. We contacted them via telephone and invited them to participate in a structured diagnostic interview using MINI‐International Neuropsychiatric Interview (MINI) (Sheehan et al., 1998) Chinese version (Si et al., 2009). A total of 244 students attended the interview and received diagnoses from certified psychiatric doctors. We screened the 244 students for eligibility using the following exclusion criteria: (a) severe depression requiring immediate pharmaceutical treatment, and/or received a major depressive disorder diagnosis with Self‐Rating Depression Scale (SDS) standard score ≥ 70; (b) diagnosed with bipolar disorder; (c) showed psychotic symptoms; (d) severely cognitively impaired; (e) currently received psychotropic or psychological intervention; (f) drug abusing; (g) other conditions that required immediate hospitalization and/or pharmaceutical treatment, such as acute suicidal attempt or severe self‐harming behaviors. A total of 189 eligible students were obtained and further invited to participate in the current suicide prevention program. A total of 97 students agreed to participate and signed the informed consent (see Figure 1). The average age of the participants was 19.20 ± 0.75 years, ranged from 17 to 21 years. 42 (43.3%) of them were male.

### 2.2 Measures

The Suicidal Behaviors Questionnaire revised (SBQ‐R) Chinese version assessed suicide behaviors and ideation (Osman et al., 2001) and functioned as the screening tool of suicidal risks. SBQ‐R has 4 items, each tapping a different dimension of suicidality. Item 1 taps into lifetime suicide ideation and/or suicide attempt. Item 2 assesses the frequency of suicidal ideation over the past 12 months. Item 3 assesses the threat of suicide attempt. Item 4 evaluates self‐reported likelihood of suicide behavior in the future. When item 1 score ≥ 2, and/or SBQ‐R total score ≥ 7, indicate heightened suicidal risks. The reliability and validity of the scale have been extensively verified in both English (Osman et al., 2001) and Chinese population (Zhao, Zhao, Xiao, Yang, & Zhang, 2013). Beck Hopelessness Scale (BHS) Chinese version was used as another indicator of suicidal risk. BHS contains 20 items and three subscales: feelings about the future, loss of motivation, and expectations (Beck, Weissman, Lester, & Trexler, 1988). The psychometric properties have been verified in Chinese samples (Liu et al., 2011). The Psychache Scale (PAS) Chinese version was used to measure the degree of psychological distress, which is another widely used scale measuring the risks of suicide (Holden, Mehta, Cunningham, & Mcleod, 2001). PAS comprises of 13 five‐points‐scaled items, with a total score ranging from 13 to 65. The reliability and validity of the Chinese version were satisfactory (Yang & Chen, 2017). Symptom Checklist‐90‐Revised (SCL‐90‐R) Chinese version was used to measure the psychopathology and psychological distress. It includes ten subscales: somatization (SOM), obsessive‐compulsive (OC), interpersonal sensitivity (INT), depression (DEP), anxiety (ANX), hostility (HOS), phobic (PHOB), paranoid ideation (PAR), psychoticism (PSY), and a category of “additional items (ADD)” (Derogatis, 1994). The reliability and validity of the Chinese version have been proven to be acceptable (Xiao, 2012). During the study period, the frequency of suicidal attempts and hospitalization due to suicidal attempts (0 = no, 1 = yes), as well as the use of psychotropic drugs (0 = no, 1 = yes) were recorded as complements to the outcome measures.

### 2.3 Intervention

We developed a 12‐week Zhong‐Yong thinking based DBT group skills training program according to the dialectical behavior therapy with Suicidal Adolescents Manual for DBT skills training group (Linehan, 1993; Miller, Rathus, & Linehan, 2007), as well as other related literatures (Gibson et al., 2014; Soler et al., 2009). The project leader designed the training content by considering: (a) the program schedule does not conflict with class schedules; (b) the program retains the essential components of regular DBT skills training. Thus, the program includes four treatment modules and a telephone‐coaching module (see Table 1). Two modules are acceptance‐oriented skills: mindfulness (2 weeks) and distress tolerance (3 weeks); two modules are change‐oriented skills: emotional regulation (4 weeks) and interpersonal effectiveness (3 weeks). Mindfulness was taught in the first two sessions and integrated throughout the whole sessions; (c) the program replaces the concept of dialectical thinking with Zhong‐Yong thinking, by using approachable language to illustrate the overarching principles of Zhong‐Yong thinking. We coached participants to develop a middle‐way (Zhong‐Yong) attitude to deal with their daily dilemmas, and to learn the wisdom of “maintaining the best balance between extremes.” We used Zhong‐Yong thinking principle to illustrate the mindfulness skills. In the distress tolerance module, we presented some prominent historical figures such as Yuanzhang Zhu and Bei Liu as role models to show how to use the doctrine of Zhong‐Yong to accept and adapt to the harsh reality without making it worse. In the emotional regulation module, we coached participants using Zhong‐Yong thinking to reevaluate the situation that might elicit extreme emotions. In the interpersonal effectiveness module, we taught participants using Zhong‐Yong thinking to cope with interpersonal conflicts, finding the middle ground in opposing perspectives. The group sizes were 7–10 members, with an average of 8 members per group. The sessions lasted 2 hr per week, lasting for 12 consecutive weeks. The therapists were one doctor‐level psychologist (a licensed professional) as the primary group leader and two graduate students as the secondary group facilitators. They were all trained previously on DBT skills. The two graduate students received DBT supervision from the doctor‐level psychologist during the study. The doctor‐level psychologist was also responsible for the adherence to treatment manuals.

**TABLE 1:** Content description of interventions in the DBT_ZYT_ and SGT group

Content description	DBT_ZYT_	SGT
Overarching theories	Zhong‐Yong (middle‐way) thinking	Interpersonal psychology
Role of the therapists	Provide psychoeducation	Provide psychoeducation
	Coach DBT skills	Foster a safe, supportive emotion expression atmosphere
	Set/review homework tasks	Facilitate effective interpersonal interactions
	Reinforce skilled behaviors	Establish group rules and limits
	Promote effective coping strategies	Prompt peer‐peer support
	Establish group rules and limits	Manage group conflict and mediate tension between group members
	Prompt peer‐peer support	
	Manage group conflict	
Mindfulness	Goals of mindfulness	Not included
	Skills for mindfulness Wise Mind What Skills How Skills Mindful breathing skills	
Distress tolerance	Explain principles of Zhong‐Yong thinking and distress tolerance	Not included
	Crisis survival skills Distraction Self‐soothing IMPROVE the moment TIP Skills	
	Reality acceptance skills Radical acceptance Turning the mind	
Emotion regulation	Explain the relationship of Zhong‐Yong thinking and emotional regulation	Help group members to understand one's emotional process and emotion regulation strategies in interpersonal interaction
	Emotion regulation skills Understanding and naming emotions Changing emotional responses Reducing vulnerability to emotion mind	Practice effective emotion regulation skills in interpersonal interaction
		Encourage emotion expression within the group
Interpersonal effectiveness	Use Zhong‐Yong thinking to cope with interpersonal conflicts, finding the middle ground in opposite perspectives	Understand interpersonal dynamics
	Use DBT skills to maintain self‐respect and good relationships with others DEAR MAN GIVE FAST	Cope with interpersonal conflict
		Enhance social support
		Express one's needs
		Learn how to say no and how to seek help from others
Telephone coaching	Enhance learned DBT skills	Provide social support and guidance to address interpersonal problems

Abbreviations: DBT_ZYT_, Zhong‐Yong thinking based dialectical behavior therapy group skills training; SGT, supportive group therapy.

We also developed a 12‐week supportive group therapy based on the theory, research, and therapeutic practice of interpersonal psychology (Horowitz & Strack, 2012). The goal was to improve self‐regulation in a safe, supportive interpersonal context. Therefore, the program included two treatment modules that were integrated in the whole sessions: emotional regulation and interpersonal effectiveness, and one telephone‐coaching module (Table 1). The group sizes, schedule, as well as the length of therapy, were the same as DBT_ZYT_ group. The group leaders included two therapists: one doctor‐level psychologist and one graduate student, and they were all skilled in group psychotherapy. The doctor‐level psychologist was responsible for the supervision and adherence to the treatment protocol during the study.

### 2.4 Procedure

After enrollment, participants were randomized in blocks of three to either DBT_ZYT_ or SGT or WL group by a computer program. Upon assignment, all participants were instructed by the research assistant to complete the outcome measures and a demographic questionnaire. Throughout the study, the research assistant was blind to study hypotheses and participants' assignments. DBT_ZYT_ and SGT groups would receive a 12‐week intervention, respectively. WL group received no special intervention during this period. After 12 weeks, all participants received postintervention assessments and a gift. Participants in DBT_ZYT_ and SGT groups were interviewed on their views and feelings about the intervention program. They were also asked to indicate on a 1–10 scale to what extent they found the program was helpful (1 = not at all helpful, 10 = very helpful), and were encouraged to continue to practice the skills in their daily life. To maintain and enhance the effectiveness of the intervention, during the 6‐month follow‐up period, the therapist‐team continually called participants of DBT_ZYT_ and SGT groups once a month to provide individual telephone coaching. Six months later, participants in intervention groups were contacted again and received the follow‐up assessments and another gift. WL group did not attend the follow‐up assessments.

To enhance the ecological validity, during the intervention and follow‐up period, we allowed all participants to receive non‐DBT individual psychotherapy voluntarily or psychiatric services as deemed appropriate by the psychiatrist. They were also encouraged to call a 24/7 hot line when crisis arises. All the psycho‐medical intervention would be recorded as covariate variables. For intervention groups, treatment attendance was also recorded. Dropout was defined as “missing more than 3 consecutive sessions or missing a total of 4 sessions.” For all the 3 groups, failing to attend the post‐treatment and/or follow‐up reassessments was considered dropout.

### 2.5 Statistical analyses

Outcome analyses were based on the intent‐to‐treatment (ITT) model, that is, the missing data were imputed using the last observation carried forward method. This method assumes that missing values are missing completely at random, and participant's responses are basically stable from the point of dropout to trial completion, rather than declining or improving further (Molnar, Hutton, & Fergusson, 2008). In the present study, if the above assumptions were seriously violated, we would use other statistical techniques to deal with the missing values, such as multiple imputation. We conducted a series of 3 (group: DBT_ZYT_, SGT, WL) by 2 (time: PRE, POST) mixed factorial (i.e., repeated measures) ANOVAs to reveal the overall intervention effects. We also conducted a series of 2 (group: DBT_ZYT_, SGT) by 3 (time: PRE, POST, F/U) mixed factorial ANOVAs to compare the short‐term and long‐term intervention effects of DBT_ZYT_ and SGT groups. If the assumption of sphericity has not been met, the Greenhouse–Geisser correction would be applied. Paired‐sample *t* tests were conducted to examine changes in the outcome measures from pre‐ to post and to follow‐up measures. The multiple paired *t* tests with a Bonferroni correction were used to keep the Type I error at 5% overall. To measure the magnitude of differences between groups, we reported Hedges's *g* or partial η^2^ as effect size indicators.

## 3 RESULTS

### 3.1 Treatment attendance and descriptive data

As shown in Figure 1, at the post‐treatment assessments, five participants (15.2%) in the DBT_ZYT_ group, five participants (15.6%) in the SGT group, and four participants (12.5%) in the WL group dropped out of the study. At the follow‐up assessments, four participants (12.1%) in the DBT_ZYT_ group, five participants (15.6%) in the SGT group dropped out of the study. The dropout rate did not significantly differ between groups both at post‐treatment and at follow‐up assessments, all *p*s > 0.05. The noncompleters did not significantly differ from completers on sex, but differed on age: Mean_completers_ = 19.3 ± 0.7, Mean_non‐completers_ = 18.8 ± 0.8, *t* (95) = 2.28, *p* = .025, *d* = 0.67. There were also significant differences on all of the baseline assessments between completers (*n* = 83) and noncompleters (*n* = 14): for SBQ‐R score, Mean_completers_ = 9.07 ± 2.51, Mean_non‐completers_ = 10.79 ± 2.67, *t* (95) = 2.34, *p* = .021, *d* = 0.66; for BHS score, Mean_completers_ = 10.16 ± 4.27, Mean_non‐completers_ = 13.14 ± 4.02, *t* (95) = 2.44, *p* = .016, *d* = 0.72; for PAS score, Mean_completers_ = 43.55 ± 11.24, Mean_non‐completers_ = 51.00 ± 7.57, *t* (95) = 2.38, *p* = .019, *d* = 0.78; for SCL‐90‐R average score, Mean_completers_ = 1.62 ± 0.74, Mean_non‐completers_ = 2.10 ± 0.68, *t* (95) = 2.26, *p* = .026, *d* = 0.67.

During the whole study period, no participants reported attempting suicide in the DBT_ZYT_ group, two participants in the SCT group and one in the WL group reported at least one suicide attempt, the suicide attempt frequency did not significantly differ among groups, Pearson *χ* ^2^ (2) = 2.12, *p* = .347. The frequency of hospitalization due to suicidal attempts and/or serious psychopathology did not differ among groups (one participant in the DBT_ZYT_ group, one in the SCT group and zero in the WL group), Pearson *χ* ^2^ (2) = 1.01, *p* = .605. There were four participants in the DBT_ZYT_ group, four in the SCT group and five in the WL group had used psychotropic drugs. The drug usage frequency did not significantly differ among groups, Pearson *χ* ^2^ (2) = 0.21, *p* = .902. There were three participants (with an average of 2.33 sessions) in the DBT_ZYT_ group, four in the SCT group (with an average of 2.25 sessions) and eight in the WL group (with an average of 2.63 sessions) had received at least one session of non‐DBT individual psychotherapy. The frequency of sessions received did not significantly differ among groups, Pearson *χ* ^2^ (10) = 7.78, *p* = .650. The intervention sessions attended did not differ between the DBT_ZYT_ and SGT groups in the ITT sample: Mean_DBT_ = 10.21 ± 1.93, Mean_SGT_ = 9.88 ± 2.09, *t* (63) = 0.68, *p* = .502, *d* = 0.16; or among treatment completers: Mean_DBT_ = 10.86 ± 1.04, Mean_SGT_ = 10.59 ± 1.08, *t* (53) = 0.92, *p* = .361, *d* = 0.25.

Inspection of the data suggested that the distribution of all questionnaire scores were within the accepted range of ±2 skewness and kurtosis for parametric analyses (Gravetter & Wallnau, 2014). The outcome data are presented in Table 2. One‐way ANOVA and independent‐sample *t* test showed that all the baseline assessments did not significantly differ among groups. The suicidal risk measures of the WL group at pre‐ and post‐treatment did not show significant changes. Although most outcome measures in the two intervention groups showed improvements, the group differences in post‐treatment and follow‐up did not reach statistical significance. As repeated measures ANOVA could control for factors that cause variability between subjects, it is more powerful to detect changes in mean scores over three time points between groups, we reported outcome analyses using repeated measures ANOVA and paired‐sample *t* test. As the assumptions of “missing at random” and “the participant's responses remaining basically stable from the point of dropout to trial completion” were not seriously violated, we would use “last observation carried forward” method to handle missing values.

**TABLE 2:** Outcome descriptive data and difference tests for intent‐to‐treat sample (*M* ± *SD*)

Outcomes	DBT_ZYT_ (*n* = 33)	SGT (*n* = 32)	WL (*n* = 32)	*F* (2, 94) or *t* (63)	Sig.
SBQ‐R					
PRE	9.64 ± 2.87	9.03 ± 1.93	9.28 ± 2.89	0.44	.643
POST	8.21 ± 2.58	7.84 ± 2.27	8.97 ± 2.82	1.60	.208
F/U	7.82 ± 2.95	8.16 ± 2.13		−.53	.599
BHS					
PRE	10.82 ± 4.69	10.38 ± 4.29	10.56 ± 4.15	.08	.920
POST	8.58 ± 4.15	8.81 ± 4.19	10.19 ± 4.07	1.43	.245
F/U	8.52 ± 4.45	9.53 ± 4.02		−.97	.338
PAS					
PRE	43.67 ± 12.84	45.50 ± 9.77	44.75 ± 10.60	.22	.801
POST	38.48 ± 12.23	41.97 ± 9.91	45.09 ± 10.66	2.94	.058
F/U	39.18 ± 12.79	43.91 ± 10.60		−1.62	.111
SCL‐90‐R					
PRE	1.65 ± 0.83	1.71 ± 0.68	1.70 ± 0.74	.05	.954
POST	1.43 ± 0.78	1.53 ± 0.66	1.69 ± 0.74	1.05	.353
F/U	1.43 ± 0.80	1.60 ± 0.68		−.94	.350
SOM					
PRE	0.99 ± 0.79	1.15 ± 0.74	1.02 ± 0.72	.40	.672
POST	0.91 ± 0.76	1.09 ± 0.74	1.00 ± 0.71	.48	.618
F/U	0.93 ± 0.78	1.14 ± 0.74		−1.12	.266
OC					
PRE	2.04 ± 0.97	1.96 ± 0.64	2.08 ± 0.88	.16	.849
POST	1.70 ± 0.87	1.78 ± 0.66	2.07 ± 0.88	1.83	.165
F/U	1.62 ± 0.88	1.92 ± 0.66		−1.52	.134
INT					
PRE	1.94 ± 0.90	1.95 ± 0.79	1.95 ± 0.89	.00	.998
POST	1.73 ± 0.86	1.65 ± 0.74	1.95 ± 0.93	1.09	.341
F/U	1.72 ± 0.92	1.69 ± 0.77		.12	.902
DEP					
PRE	2.11 ± 1.08	2.20 ± 0.86	2.19 ± 0.95	.09	.914
POST	1.76 ± 0.97	1.97 ± 0.87	2.17 ± 0.96	1.53	.221
F/U	1.80 ± 1.00	2.01 ± 0.90		−.91	.367
ANX					
PRE	1.82 ± 0.97	1.98 ± 0.82	1.88 ± 0.97	.26	.770
POST	1.53 ± 0.86	1.78 ± 0.77	1.90 ± 0.96	1.56	.216
F/U	1.48 ± 0.88	1.86 ± 0.82		−1.81	.076
HOS					
PRE	1.44 ± 1.15	1.47 ± 1.03	1.81 ± 0.97	1.26	.290
POST	1.33 ± 1.07	1.24 ± 0.99	1.74 ± 0.71	2.34	.102
F/U	1.34 ± 1.08	1.31 ± 1.03		.10	.922
PHOB					
PRE	1.29 ± 1.07	1.25 ± 0.94	1.08 ± 0.83	.45	.637
POST	1.18 ± 1.03	1.05 ± 0.85	1.10 ± 0.86	.17	.841
F/U	1.17 ± 1.03	1.15 ± 0.93		.09	.931
PAR					
PRE	1.46 ± 0.93	1.60 ± 0.97	1.59 ± 0.83	.23	.797
POST	1.28 ± 0.89	1.44 ± 0.96	1.61 ± 0.85	1.10	.336
F/U	1.32 ± 0.90	1.52 ± 0.98		−.87	.390
PSY					
PRE	1.58 ± 0.78	1.59 ± 0.71	1.53 ± 0.72	.05	.951
POST	1.39 ± 0.72	1.43 ± 0.71	1.51 ± 0.70	.24	.787
F/U	1.38 ± 0.75	1.54 ± 0.73		−.83	.408
ADD					
PRE	1.61 ± 1.03	1.60 ± 0.88	1.68 ± 0.97	.06	.938
POST	1.37 ± 0.95	1.47 ± 0.81	1.71 ± 0.97	1.16	.317
F/U	1.40 ± 0.99	1.55 ± 0.81		−.69	.491

Abbreviations: ADD, additional items subscale average score; ANX, anxiety subscale average score; BHS, Beck Hopelessness Scale total score; DBT_ZYT_, Zhong‐Yong thinking based dialectical behavior therapy group skills training; DEP, depression subscale average score; F/U, 6‐month follow‐up assessment; HOS, hostility subscale average score; INT, interpersonal sensitivity subscale average score; OC, obsessive‐compulsive subscale average score; PAR, paranoid ideation subscale average score; PAS, Psychache Scale total score; PHOB, phobic subscale average score; POST, post‐treatment assessment; PRE, pretreatment assessment; PSY, psychoticism subscale average score; SBQ‐R, Suicidal Behaviors Questionnaire revised total score; SCL‐90‐R, Symptom Checklist‐90‐revised total average score; SGT, supportive group therapy; SOM, somatization subscale average score; WL, wait list.

### 3.2 Outcome analyses at post‐treatment measures

As shown in Table 3, both intervention groups evidenced more reductions in post‐treatment measures compared with the WL group. Repeated measures ANOVAs of group (DBT_ZYT_, SGT, WL) × time (PRE, POST) showed similar patterns for suicidal behaviors and hopelessness, as well as psychological distress and psychopathological symptoms. All analyses demonstrated significant main effects of time and time by group interaction, but no significant main effect of group. Paired‐sample *t* tests indicated that in the DBT_ZYT_ and SGT group, all outcome measures significantly reduced from PRE to POST, but no significantly change was observed in the WL group. All subscales of SCL‐90‐R, except for SOM, revealed significant main effects of time and time by group interaction (see Table 4). Most effect sizes were comparable between the DBT_ZYT_ and SGT groups, and the SGT group showed slightly larger effect sizes for the INT, HOS, and PHOB subscales (the differences of Hedges'g > 0.08).

**TABLE 3:** Repeated measures ANOVAs at pre‐ and post‐treatment and follow‐up of the total scores of outcome measures

	DBT_ZYT_ (*n* = 33)	SGT (*n* = 32)	WL (*n* = 32)	Time effect (*F*; *p* value; η*_p_* ^2^)	Time × group effect (*F*; *p* value; η*_p_* ^2^)	Group effect (*F*; *p* value; η*_p_* ^2^)
SBQ‐R						
Pre–post ES [95% CI] and *p* value	0.50 [0.29, 0.75] *p* < .001	0.54 [0.27, 0.85] *p* < .001	0.11 [−0.05, 0.27] *p* = .169	*F* (1, 94) = 44.23; *p* < .001; η*_p_* ^2^ = 0.32	*F* (2, 94) = 5.32; *p* = .006; η*_p_* ^2^ = 0.10	*F* (2, 94) = .65; *p* = .525; η*_p_* ^2^ = 0.01
Post‐F/U ES [95% CI] and *p* value	0.13 [−0.04, 0.32] *p* = .113	−0.14 [−0.36, 0.07] *p* = .209		*F* (2, 126) = 31.19; *p* < .001; η*_p_* ^2^ = 0.33	*F* (2, 126) = 3.20; *p* = .044; η*_p_* ^2^ = 0.05	*F* (1, 63) = .14; *p* = .714; η*_p_* ^2^ < 0.01
BHS						
Pre–post ES [95% CI] and *p* value	0.48 [0.30, 0.70] *p* < .001	0.36 [0.19, 0.54] *p* < .001	0.09 [−0.06, 0.24] *p* = .245	*F* (1, 94) = 54.05; *p* < .001; η*_p_* ^2^ = 0.37	*F* (2, 94) = 8.31; *p* < .001; η*_p_* ^2^ = 0.15	*F* (2, 94) = .33; *p* = .717; η*_p_* ^2^ < 0.01
Post‐F/U ES [95% CI] and *p* value	0.01 [−0.10, 0.12] *p* = .797	−0.17 [−0.29, −0.06] *p* = .004		*F* (1.6, 101.8) = 40.60; *p* < .001; η*_p_* ^2^ = 0.39	*F* (1.6, 101.8) = 5.24; *p* = .011; η*_p_* ^2^ = 0.08	*F* (1, 63) = .07; *p* = .795; η*_p_* ^2^ < 0.01
PAS						
Pre–post ES [95% CI] and *p* value	0.40 [0.26, 0.57] *p* < .001	0.35 [0.20, 0.51] *p* < .001	−0.03 [−0.19, 0.12] *p* = .686	*F* (1, 94) = 41.34; *p* < .001; η*_p_* ^2^ = 0.31	*F* (2, 94) = 14.26; *p* < .001; η*_p_* ^2^ = 0.23	*F* (2, 94) = 1.07; *p* = .347; η*_p_* ^2^ = 0.02
Post‐F/U ES [95% CI] and *p* value	−0.05 [−0.15, 0.04] *p* = .285	−0.18 [−0.31, −0.07] *p* = .004		*F* (1.8, 114.3) = 38.16; *p* < .001; η*_p_* ^2^ = 0.38	*F* (2, 126) = 4.02; *p* = .024; η*_p_* ^2^ = 0.06	*F* (1, 63) = 1.45; *p* = .233; η*_p_* ^2^ = 0.02
SCL‐90‐R						
Pre–post ES [95% CI] and *p* value	0.25 [0.17, 0.37] *p* < .001	0.26 [0.18, 0.35] *p* < .001	0.01 [−0.04, 0.06] *p* = .787	*F* (1, 94) = 99.22; *p* < .001; η*_p_* ^2^ = 0.51	*F* (2, 94) = 23.18; *p* < .001; η*_p_* ^2^ = 0.33	*F* (2, 94) = .34; *p* = .711; η*_p_* ^2^ < 0.01
Post‐F/U ES [95% CI] and *p* value	0.00 [−0.05, 0.05] *p* = .871	−0.10 [−0.16, −0.05] *p* < .001		*F* (1.5, 94.8) = 94.93; *p* < .001; η*_p_* ^2^ = 0.60	*F* (1.5, 94.8) = 7.49; *p* = .003; η*_p_* ^2^ = 0.11	*F* (1, 63) = .33; *p* = .568; η*_p_* ^2^ < 0.01

Note

Abbreviations: BHS, Beck Hopelessness Scale total score; DBT_ZYT_, Zhong‐Yong thinking based dialectical behavior therapy group skills training; ES, effect size (Hedges'g); F/U, 6‐month follow‐up assessment; PAS, Psychache Scale total score; POST, post‐treatment assessment; PRE, pretreatment assessment; SBQ‐R, Suicidal Behaviors Questionnaire revised total score; SCL‐90‐R, Symptom Checklist‐90‐revised total average score; SGT, supportive group therapy; WL, wait list.

**TABLE 4:** Repeated measures ANOVAs at pre‐ and post‐treatment and follow‐up of the SCL‐90‐R subscale scores

	DBT_ZYT_ (*n* = 33)	SGT (*n* = 32)	WL (*n* = 32)	Time effect (*F*; *p* value; η*_p_* ^2^)	Time × Group Effect (*F*; *p* value; η*_p_* ^2^)	Group Effect (*F*; *p* value; η*_p_* ^2^)
SOM						
Pre–post ES [95% CI] and *p* value	0.10 [0.05, 0.16] *p* < .001	0.08 [0.03, 0.13] *p* = .002	−0.03 [−0.12, 0.05] *p* = .440	*F* (1, 94) = 22.54; *p* < .001; η*_p_* ^2^ = 0.19	*F* (2, 94) = 2.93; *p* = .058; η*_p_* ^2^ = 0.06	*F* (2, 94) = .42; *p* = .656; η*_p_* ^2^ < 0.01
Post‐F/U ES [95% CI] and *p* value	−0.02 [−0.08, 0.02] *p* = .247	−0.07 [−0.12, −0.02] *p* = .004		*F* (2, 126) = 15.78; *p* < .001; η*_p_* ^2^ = 0.20	*F* (2, 126) = 2.06; *p* = .132; η*_p_* ^2^ = 0.03	*F* (1, 63) = .95; *p* = .335; η*_p_* ^2^ = 0.02
OC						
Pre–post ES [95% CI] and *p* value	0.34 [0.23, 0.50] *p* < .001	0.27 [0.17, 0.39] *p* < .001	0.01 [−0.06, 0.08] *p* = .835	*F* (1, 94) = 62.65; *p* < .001; η*_p_* ^2^ = 0.40	*F* (2, 94) = 19.44; *p* < .001; η*_p_* ^2^ = 0.29	*F* (2, 94) = .65; *p* = .523; η*_p_* ^2^ = 0.01
Post‐F/U ES [95% CI] and *p* value	0.09 [0.04, 0.14] *p* = .002	−0.21 [−0.30, −0.12] *p* < .001		*F* (1.4, 87.7) = 50.71; *p* < .001；η*_p_* ^2^ = 0.45	*F* (1.4, 87.7) = 23.12; *p* < .001; η*_p_* ^2^ = 0.27	*F* (1, 63) = .26; *p* = .610; η*_p_* ^2^ < 0.01
INT						
Pre–post ES [95% CI] and *p* value	0.28 [0.15, 0.33] *p* < .001	0.37 [0.26, 0.52] *p* < .001	0.00 [−0.09, 0.09] *p* = .928	*F* (1, 94) = 64.61; *p* < .001; η*_p_* ^2^ = 0.41	*F* (2, 94) = 16.85; *p* < .001; η*_p_* ^2^ = 0.26	*F* (2, 94) = .29; *p* = .751; η*_p_* ^2^ < 0.01
Post‐F/U ES [95% CI] and *p* value	0.01 [−0.06, 0.08] *p* = .653	−0.05 [−0.15, 0.05] *p* = .235		*F* (2, 126) = 57.07; *p* < .001; η*_p_* ^2^ = 0.48	*F* (2, 126) = 1.47; *p* = .234; η*_p_* ^2^ = 0.02	*F* (1, 63) = .03; *p* = .864; η*_p_* ^2^ < 0.01
DEP						
Pre–post ES [95% CI] and *p* value	0.32 [0.20, 0.48] *p* < .001	0.26 [0.16, 0.37] *p* < .001	0.02 [−0.06, 0.11] *p* = .537	*F* (1, 94) = 54.05; *p* < .001; η*_p_* ^2^ = 0.37	*F* (2, 94) = 12.08; *p* < .001; η*_p_* ^2^ = 0.20	*F* (2, 94) = .55; *p* = .576; η*_p_* ^2^ = 0.01
Post‐F/U ES [95% CI] and *p* value	−0.04 [−0.09, 0.01] *p* = .195	−0.04 [−0.12, 0.03] *p* = .217		*F* (1.6, 99.6) = 50.13; *p* < .001; η*_p_* ^2^ = 0.44	*F* (1.6, 99.6) = 2.41; *p* = .107; η*_p_* ^2^ = 0.04	*F* (1, 63) = .56; *p* = .457; η*_p_* ^2^ = 0.01
ANX						
Pre–post ES [95% CI] and *p* value	0.29 [0.17, 0.46] *p* < .001	0.23 [0.15, 0.35] *p* < .001	−0.02 [−0.09, 0.05] *p* = .402	*F* (1, 94) = 39.97; *p* < .001; η*_p_* ^2^ = 0.30	*F* (2, 94) = 14.97; *p* < .001; η*_p_* ^2^ = 0.24	*F* (2, 94) = .61; *p* = .544; η*_p_* ^2^ = 0.01
Post‐F/U ES [95% CI] and *p* value	0.06 [0.00, 0.11] *p* = .054	−0.09 [−0.18, −0.02] *p* = .006		*F* (1.5, 92.3) = 43.26; *p* < .001; η*_p_* ^2^ = 0.41	*F* (1.5, 92.3) = 6.95; *p* = .004; η*_p_* ^2^ = 0.10	*F* (1, 63) = 1.59; *p* = .212; η*_p_* ^2^ = 0.03
HOS						
Pre–post ES [95% CI] and *p* value	0.09 [0.04, 0.16] *p* = .001	0.22 [0.14, 0.32] *p* < .001	0.06 [−0.07, 0.23] *p* = .223	*F* (1, 94) = 32.74; *p* < .001; η*_p_* ^2^ = 0.26	*F* (2, 94) = 4.27; *p* = 0.017; η*_p_* ^2^ = 0.08	*F* (2, 94) = 1.73; *p* = .182; η*_p_* ^2^ = 0.04
Post‐F/U ES [95% CI] and *p* value	−0.01 [−0.06, 0.04] *p* = .839	−0.07 [−0.12, −0.02] *p* = .024		*F* (1.7, 106.1) = 27.24; *p* < .001; η*_p_* ^2^ = 0.30	*F* (1.7, 106.1) = 3.37; *p* = .046; η*_p_* ^2^ = 0.05	*F* (1, 63) = .01; *p* = .909; η*_p_* ^2^ < 0.01
PHOB						
Pre–post ES [95% CI] and *p* value	0.10 [0.05, 0.16] *p* < .001	0.19 [0.13, 0.32] *p* < .001	−0.02 [−0.11, 0.06] *p* = .544	*F* (1, 94) = 27.87; *p* < .001; η*_p_* ^2^ = 0.23	*F* (2, 94) = 13.08; *p* < .001; η*_p_* ^2^ = 0.22	*F* (2, 94) = .20; *p* = .817; η*_p_* ^2^ < 0.01
Post‐F/U ES [95% CI] and *p* value	0.01 [−0.04, 0.06] *p* = .690	−0.10 [−0.19, −0.03] *p* = .004		*F* (2, 126) = 26.94; *p* < .001; η*_p_* ^2^ = 0.30	*F* (2, 126) = 4.07; *p* = .019; η*_p_* ^2^ = 0.06	*F* (1, 63) = .07; *p* = .800; η*_p_* ^2^ < 0.01
PAR						
Pre–post ES [95% CI] and *p* value	0.19 [0.11, 0.28] *p* < .001	0.16 [0.10, 0.23] *p* < .001	−0.02 [−0.11, 0.06] *p* = .675	*F* (1, 94) = 33.11; *p* < .001; η*_p_* ^2^ = 0.26	*F* (2, 94) = 11.01; *p* < .001; η*_p_* ^2^ = 0.19	*F* (2, 94) = .55; *p* = .580; η*_p_* ^2^ = 0.01
Post‐F/U ES [95% CI] and *p* value	−0.04 [−0.11, 0.02] *p* = .187	−0.08 [−0.14, −0.03] *p* = .011		*F* (2, 126) = 31.54; *p* < .001; η*_p_* ^2^ = 0.33	*F* (2, 126) = 1.22,; *p* = .300; η*_p_* ^2^ = 0.02	*F* (1, 63) = .52; *p* = .472; η*_p_* ^2^ < 0.01
PSY						
Pre–post ES [95% CI] and *p* value	0.23 [0.17, 0.35] *p* < .001	0.22 [0.14, 0.31] *p* < .001	0.03 [−0.06, 0.11] *p* = .391	*F* (1, 94) = 62.64; *p* < .001; η*_p_* ^2^ = 0.40	*F* (2, 94) = 10.54; *p* < .001; η*_p_* ^2^ = 0.18	*F* (2, 94) = .02; *p* = .978; η*_p_* ^2^ < 0.01
Post‐F/U ES [95% CI] and *p* value	0.01 [−0.04, 0.06] *p* = .879	−0.15 [−0.21, −0.09] *p* < .001		*F* (1.7, 109.4) = 47.25; *p* < .001; η*_p_* ^2^ = 0.43	*F* (1.7, 109.4) = 8.52; *p* = .001; η*_p_* ^2^ = 0.12	*F* (1, 63) = .14; *p* = .707; η*_p_* ^2^ < 0.01
ADD						
Pre–post ES [95% CI] and *p* value	0.21 [0.16, 0.32] *p* < .001	0.14 [0.07, 0.24] *p* < .001	−0.03 [−0.12, 0.05] *p* = .526	*F* (1, 94) = 30.83; *p* < .001; η*_p_* ^2^ = 0.25	*F* (2, 94) = 14.17; *p* < .001; η*_p_* ^2^ = 0.23	*F* (2, 94) = .42; *p* = .656; η*_p_* ^2^ < 0.01
Post‐F/U ES [95% CI] and *p* value	−0.03 [−0.08, 0.02] *p* = .315	−0.10 [−0.17, −0.03] *p* = .010		*F* (1.7, 108.3) = 30.15; *p* < .001; η*_p_* ^2^ = 0.32	*F* (1.7, 108.3) = 5.58; *p* = .007; η*_p_* ^2^ = 0.08	*F* (1, 63) = .15; *p* = .705; η*_p_* ^2^ < 0.01

Abbreviations: ADD, additional items subscale average score; ANX, anxiety subscale average score; DBT_ZYT_, Zhong‐Yong thinking based dialectical behavior therapy group skills training; DEP, depression subscale average score; ES, effect size (Hedges'g); F/U, 6‐month follow‐up assessment; HOS, hostility subscale average score; INT, interpersonal sensitivity subscale average score; OC, obsessive‐compulsive subscale average score; PAR, paranoid ideation subscale average score; PHOB, phobic subscale average score; POST, post‐treatment assessment; PRE, pretreatment assessment; PSY, psychoticism subscale average score; SGT, supportive group therapy; SOM, somatization subscale average score; WL, wait list.

### 3.3 Outcome analyses at follow‐up measures

Since only the two intervention groups were assessed at follow‐up, we conducted separate 2 (group: DBT_ZYT_, SGT) by 3 (time: PRE, POST, F/U) repeated measures ANOVAs for four outcome measures to detect the group differences across the three time points. As shown in Table 3 (with gray shading), all analyses demonstrated significant main effects of time and time by group interaction, but no significant main effect of group, indicating the two intervention groups evidenced different changes across the three time points. Paired‐sample *t* tests indicated that in the DBT_ZYT_ group all the outcome measures did not significantly change from POST to F/U. In the SGT group, however, the BHS, PAS, and SCL‐90‐R average score significantly increased from POST to F/U.

To further explore the differential effects of the two interventions programs on psychopathological symptoms, we conducted separate 2 (group: DBT_ZYT_, SGT) by 3 (time: PRE, POST, F/U) repeated measures ANOVAs for ten subscales of SCL‐90‐R (Table 4 in gray shading). There were significant main effects of time in all ten subscales. The interaction effects of time by group were not significant for the SOM, INT, DEP, and PAR subscale, indicating the two intervention groups had similar effects on somatization, interpersonal sensitivity, depression, and paranoid ideation symptoms. As for the OC, ANX, HOS, PHOB, PSY, and ADD subscales, the effects of time by group interaction were significant. Paired‐sample *t* tests revealed that in the DBT_ZYT_ group, the improvement of obsessive compulsive, anxiety, hostility, phobic, psychotic, and additional symptoms remained stable or even enhanced from POST to F/U. However, in the SGT group, the above‐mentioned subscale scores significantly increased from POST to F/U.

## 4 DISCUSSION

In the present study, we found that the two intervention groups evidenced more reductions in suicidal risks at post‐treatment measures compared with the wait‐list group. Specifically, at post‐treatment, the levels of suicidal ideation, hopelessness, psychache symptoms, and general psychopathology significantly decreased in both intervention groups. More importantly, the changes observed during intervention were better maintained in the DBT_ZYT_ group rather than in the SGT group. In another word, the short‐term treatment efficacy was basically comparable between the two intervention groups, but Zhong‐Yong thinking based DBT group skills training demonstrated superior efficacy compared with supportive group therapy in the long term. In particular, the two interventions had similar effects on participants’ short‐ and long‐term somatization, depressive, and paranoid ideation symptoms. However, DBT_ZYT_ was more effective in improving participants’ long‐term obsessive compulsive, anxiety, hostility, phobic, psychotic, and additional symptoms; while supportive group therapy showed slightly superior efficacy in improving participants’ short‐term interpersonal sensitivity, hostility, and phobic symptoms. We speculate that reductions in psychopathological symptoms as measured by SCL‐90‐R may be critical for the long‐term maintenance of reduction in suicidal behaviors. Therefore, these findings provided empirical evidence for designing the most suitable psycho‐behavioral intervention programs for different subgroups of high‐risk suicidal young adults with diverse psychopathological profiles and cultural backgrounds.

As both intervention programs showed significant short‐term effectiveness in reducing suicidal ideation, hopelessness, psychache, and psychopathological symptoms, some nonspecific factors (e.g., social support, therapeutic alliance, and the structure of treatment) may contribute to the treatment effects. However, in the following section, we will focus our discussion on the differences in long‐term efficacy between the two interventions to reveal the mechanisms by which psycho‐behavioral interventions might work in suicide prevention programs.

Previous studies demonstrated that compared with older adults, young adults often experienced more intense psychological distress (Brummer, Stopa, & Bucks, 2014) and greater emotion regulation difficulties (Orgeta, 2009). These can lead to impulsive emotional responses, such as suicidal ideation and attempts. Studies have shown that DBT skills training could address the four main areas in which youth typically have problems: difficulty of managing emotions, confusion about self, impulsive behaviors, and interpersonal problems in school setting (Mazza et al., 2016). Compared to SGT, DBT skills training is more structured and places more emphasis on psychoeducation, skills training, and the active role of the therapist (Soler et al., 2009). Previous studies demonstrated that skills training is an essential element in suicide prevention programs in view of the emotion regulation deficit prominent in suicidal young adults (Robinson, Hetrick, & Martin, 2011). In the DBT_ZYT_ program, we coached participants a set of distress tolerance skills to manage their emotional distress and suicidal urges, the beneficial effects were testified by the observable decrease of hopelessness and psychache scores. Furthermore, we coached participants mindfulness skills in the first module of the DBT_ZYT_ program, this skill is very important and functions as the basis for the subsequent training modules in reducing participants’ psychological distress and psychopathological symptoms. Previous studies have suggested that using mindfulness as the primary intervention, or using mindfulness skills as an indispensable part of the intervention program is essential for improving the psychological well‐being of college students (Chen, Yang, Wang, & Zhang, 2013; Yang et al., 2018).

Our findings demonstrate that Zhong‐Yong thinking could facilitate distress tolerance. The goal of distress tolerance is to get through the painful moment without creating a bigger problem (McKay, Wood, & Brantley, 2019). However, for most westerners, the idea of sitting with discomfort and not trying to “fix” the problem could be a challenging concept. We found that distress tolerance skills were readily accepted and well‐practiced by those high Zhong‐Yong thinkers. Zhong‐Yong thinking could help participants to endure the present pain, just as the Analects of Confucius put it, “A little Impatience Spoils Great Plans.” Those high middle‐way thinkers believed that enduring short‐term discomfort could lessen long‐term suffering, and the present misfortune might be a blessing in disguise. These beliefs could help them find out positive meanings out of the present pain. Similar to dialectical thinking, Zhong‐Yong thinking discourages “black‐or‐white” and “all‐or‐nothing” thinking, and promotes holistic thinking (Yang et al., 2016). Zhong‐Yong thinking also advocates accepting different perspectives from others, seeking harmony and consistency, not simply compromising between opposite perspectives (Wu & Lin, 2005). These thinking styles are beneficial for coping with interpersonal conflicts, finding the middle ground in opposite perspectives and facilitating interpersonal effectiveness. The interview revealed that DEARMAN skill was difficult to learn for those depressed young adults. In the first half of the DBT_ZYT_ program, they were hard to keep an assertive, confident, eye‐contacted position when they expressed a need and tended to be too apologetic or confrontational when they declined someone's request. However, in the later stage of the program, most participants in the DBT_ZYT_ group gradually learned how to find a middle‐wayed manner to express their need or to decline unreasonable requests. We thus conclude that in a population traditionally dictated by Confucian culture, Zhong‐Yong thinking not only could integrate with standardized DBT skills training, but also has special strength in enhancing DBT’s efficacy.

The current study has some notable strengths. For example, we recruited a subclinical sample with a per group sample size large enough to detect a reliable effect; we used a rigorous randomized, controlled design with a supportive group and wait‐list group to test the efficacy of DBT_ZYT_; we conducted 6‐month follow‐up measures in the two intervention groups to examine their long‐term effects. There were also several limitations should be noted, which temper the conclusions could be made and also recommend directions for future study. First, the sample was heterogeneous with diverse diagnoses and some of the participants did not meet any mental disorder diagnosis, leading to the limit that the conclusion may be difficult to generalize to a wider population. Second, only the research assistant who administered the outcome measures was blind to study hypotheses and participants' assignments, it was difficult to keep the participants as well as the therapists blind to study hypotheses, therefore, we are unable to rule out the possible placebo effect from the true intervention efficacy. Third, the wait‐list group did not receive the 6‐month follow‐up assessment, there might be a natural rate of improvement due to possible assessment reactivity. Fourth, we have failed to provide adherence ratings for either treatment groups. Thus, we cannot definitively state that the treatment was in strict compliance with the manuals. The last limitation was related to the reliance on self‐report outcomes, which may attenuate the validity of causal conclusions due to social desirability effect, inadequate introspective ability, and other response biases. Future studies could use more rigorous double‐blind design to verify the conclusions in a wider population, including clinical and subclinical populations, and at the meantime combine self‐report outcomes with other information, such as behavior or physiological data. Using multi‐modal assessments could provide a more global and accurate picture of the participants.

To conclude, compared to supportive group therapy, Zhong‐Yong thinking based dialectical behavior therapy demonstrated comparable short‐term efficacy in reducing Chinese college students’ suicidal risks; at the 6‐month follow‐up, the therapeutic gains were better maintained in the DBT_ZYT_ group rather than in the SGT group. Zhong‐Yong thinking not only could integrate with DBT skills training in a Chinese young adult population, but also has special strength in enhancing DBT’s efficacy.

## CONFLICT OF INTEREST

The authors declare no potential conflict of interest.

## AUTHOR CONTRIBUTION

Xueling Yang, Ding Liu, and Xiaoyuan Zhang designed the study and the intervention procedure; You Wang, Yu Chen, Weichen Chen, Caiyan Yang, Peining Zhang, and Siyuan Ding participated in the intervention and collected the data; Xiaoyuan Zhang supervised the intervention protocol; Xueling Yang, Ding Liu, and You Wang analyzed the data and wrote the manuscript. All authors contributed to and have approved the final manuscript.

## Data Availability

The data that support the findings of this study are openly available at https://data.mendeley.com/datasets/pgvzk9sjzx/3
